# Deformability limits of *Plasmodium falciparum*-infected red blood cells

**DOI:** 10.1111/j.1462-5822.2009.01334.x

**Published:** 2009-05-26

**Authors:** Thurston Herricks, Meher Antia, Pradipsinh K Rathod

**Affiliations:** Department of Chemistry, University of WashingtonSeattle, WA 98195, USA

## Abstract

Splenic filtration of infected red blood cells (RBCs) may contribute to innate immunity and variable outcomes of malaria infections. We show that filterability of individual RBCs is well predicted by the minimum cylindrical diameter (MCD) which is calculated from a RBC's surface area and volume. The MCD describes the smallest diameter tube or smallest pore that a cell may fit through without increasing its surface area. A microfluidic device was developed to measure the MCD from thousands of individual infected RBCs (IRBCs) and uninfected RBCs (URBCs). Average MCD changes during the blood-stage cycle of *Plasmodium falciparum* were tracked for the cytoadherent strain ITG and the knobless strain Dd2. The MCD values for IRBCs and URBCs raise several new intriguing insights into how the spleen may remove IRBCs: some early-stage ring-IRBCs, and not just late-stage schizont-IRBCs, may be highly susceptible to filtration. In addition, knobby parasites may limit surface area expansions and thus confer high MCDs on IRBCs. Finally, URBCs, in culture with IRBCs, show higher surface area loss which makes them more susceptible to filtration than naive URBCs. These findings raise important basic questions about the variable pathology of malaria infections and metabolic process that affect volume and surface area of IRBCs.

## Introduction

The pathology of severe and complicated malaria infections is highly unpredictable and often fatal (Miller *et al*., 1994). The factors contributing to variable outcomes of malaria infection remain poorly understood, with some infected individuals exhibiting high parasitaemia without clinical complications and others becoming severely ill at much lower levels of parasitaemia. Complex changes in the physical and biochemical properties of infected red blood cells (IRBCs) both contribute to disease symptoms and help the parasite avoid the host's defences. For instance, it is widely accepted that mature forms of IRBCs exhibit reduced deformability, which contributes to entrapment and clearance by the spleen. Parasites are believed to have evolved the ability to cytoadhere to tissue-specific ligands in order to avoid splenic clearance (David *et al*., 1983; Clark *et al*., 2006). Cytoadherence and loss of IRBC deformability are also associated with capillary blockage and organ failure (Turner, 1997). For these reasons, a large body of experimental work has been devoted to measuring the deformability of IRBCs and discerning the pathology of severe malaria infections (George *et al*., 1967; Miller *et al*., 1971; Cranston *et al*., 1984; Areekul and Yamarat, 1988; Paulitschke and Nash, 1993; Dondorp *et al*., 1997; 2002; Nagao *et al*., 2000; Glenister *et al*., 2002; Suwanarusk *et al*., 2004; Mills *et al*., 2007; Safeukui *et al*., 2008).

Clearance of foreign and damaged red blood cells (RBCs) by the spleen occurs through two primary mechanisms: a physical selection and an immune-mediated recognition followed by their phagocytosis (Groom *et al*., 1991; Moghimi, 1995). Physical selection of IRBCs or damaged RBCs in the spleen has been related to their reduced deformability which can be measured using a variety of techniques (Miller *et al*., 1971; Schnitzer *et al*., 1972; Areekul and Yamarat, 1988). Ektacytometry measurements reported that IRBCs have a reduced deformability index, which is the ratio of a cell's length to width under a fluid sheer stress (Cranston *et al*., 1984; Dondorp *et al*., 1997). Ektacytometry has also shown the extent to which the deformability is altered and has offered a potential prognosis on the outcome of some severe malaria infections (Dondorp *et al*., 2002). Additionally, micropipette studies have described changes in deformability of IRBCs or damaged RBCs as an increase in the cell membrane's stiffness (Paulitschke and Nash, 1993; Glenister *et al*., 2002). Blood from monkeys infected with *Plasmodium knowlesi* passed through polycarbonate sieves also showed altered rheological properties, which was attributed to interactions between IRBCs and uninfected RBCs (URBCs) as well as decreased deformability of the parasitized cells (Miller *et al*., 1971). Recently, studies that stretched IRBCs using optical traps have also reported increased elastic modulus or stiffness of the cell (Mills *et al*., 2007).

Despite these important early studies, the relationship between *in vitro* measurements of altered IRBC deformability and the varied outcomes of malaria pathogenesis remains unclear. First, it is not entirely clear whether variations in deformability, under limited stresses, are relevant measurements for predicting circulatory behaviour of IRBCs. The shear stresses that cells experience under physiological conditions are of the same magnitude as the elastic modulus of IRBCs and URBCs (Paulitschke and Nash, 1993; Dondorp *et al*., 1997; Pries and Secomb, 2000; Glenister *et al*., 2002). If the forces under physiological conditions are always sufficient to deform a cell, then the intrinsic limits to which a cell can deform is more relevant to describing RBC survival in circulation than a two- to threefold increase in a cell's elastic modulus. Second, most studies that measure changes in IRBC deformability are unable to distinguish between the underlying structural factors that contribute to these changes. Cell deformability is primarily determined by three factors: the surface area and volume of the cell, the membrane viscoelasticity and the cytoplasmic viscosity (Fung, 1966). Few studies to date have identified which of these factors is most important in contributing to loss of IRBC deformability. Furthermore no study has determined which of the above properties might cause RBC obstruction of small capillaries or RBC entrapment in the spleen that are thought to cause disease complications. Micropipette aspiration has been used to obtain information on these contributing factors to IRBC deformability (Nash *et al*., 1989). However, the study concluded that the underlying reasons for loss of RBC deformability were complicated, and varied according to the parasite's stage of development. For instance, in rings, the surface area to volume played the largest role, while in trophozoites and schizonts the presence of a large, rigid parasite in the RBC cytoplasm led to loss of deformability. While micropipette measurements were the first to show that the surface area and volume of individual IRBCs could be crucial to understanding the origins of RBC deformability, this method is labour intensive and thus cannot provide the data required to obtain population variance of malaria infections between patients with different disease outcomes. Ektacytometry uses an interference pattern to measure the deformability index, a ratio of the average length and average width of RBCs under shear stresses. While ektacytometry has an advantage of measuring the bulk properties of cells under a wide variety of shear stresses, the disadvantage is that information on the underlying geometry, such as the variation of absolute cell length and width, is not easily derived from the deformability index measurement. RBCs of differing sizes may have the same length to width proportion and hence an identical deformability index. The ability of ektacytometry to measure a RBC's stress-strain response is a true advantage; this method does not make absolute measurements of an individual RBC's geometry which ultimately limits the extent that a cell can deform.

The present study adapted a microfluidic technique which was previously used to accurately measure the surface area and volume of thousands of individual normal RBCs (Gifford *et al*., 2003). These measurements were used to calculate a geometric parameter, the minimum cylindrical diameter (MCD), which describes the smallest diameter tube a cell may pass through without increasing its surface area or lysing (Rand, 1964; Canham and Burton, 1968). The equivalent to a small tube that limits the ‘size’ of circulating cells may be encountered as cells pass through the reticular mesh and interendothelial slits of the spleen (Canham and Burton, 1968). For a given cell, the MCD may then describe a criterion that determines whether IRBCs may be physically segregated and trapped by the spleen, or contribute to obstruction of capillaries in the microcirculation. By extension, the MCD population distribution of RBCs circulating in a patient is hypothesized to be related to an individual's splenic function. While these arguments relating a RBC's geometry to its filterability were originally made to explain the relationship between the spleen and a condition called spherocytic anaemia (Canham and Burton, 1968), these concepts have great potential for understanding how the spleen recognizes parasite-induced changes in RBCs.

The microfluidic system presented here was used to study the deformability limits of populations of IRBCs and URBCs in culture. The resulting MCD measurements of RBCs are specific to the cell's geometry and do not depend on the parameters of cell viscosity and membrane rigidity which can be measured using other well-established methods. The surface area and volume measurements from the microfluidic devices can quantitatively predict the ability of IRBCs and URBCs to pass through filters and microfluidic constrictions. These predictions were experimentally verified in arrays of microchannel constrictions with precisely engineered dimensions and in bulk passage through filters with well-defined pore size. The results show that cell geometry is critical to cell filtration and that microfluidic tools that measure populations of RBCs will be important to understand splenic management of malaria infections. This analytical system is also expected to help illuminate changes in physiology relative to parasite development as well as to evaluate parameters important in patient-to-patient variations in the severity of malaria infections.

## Results

### General approach for microfluidic measurements and evaluation of measurements

Previous experiments have validated the use of microfluidic devices, coupled with batch image processing algorithms, to accurately measure the surface area and volume measurements of a large number of individual RBCs (Gifford *et al*., 2003; 2006). In this work, a microfluidic system was custom designed to accommodate the large variation in cell volume due to the presence of malaria parasites within the RBC. A batch image-processing algorithm was developed to specifically measure thousands of individual IRBCs and URBCs. In order to make meaningful interpretations in a background of natural variability in surface area and volume, MCD was calculated as a metric for comparison of cells. MCD histograms from URBCs and IRBCs allowed quantitative tracking of parasite-induced alterations to populations of cells over time.

**Fig. 1:**
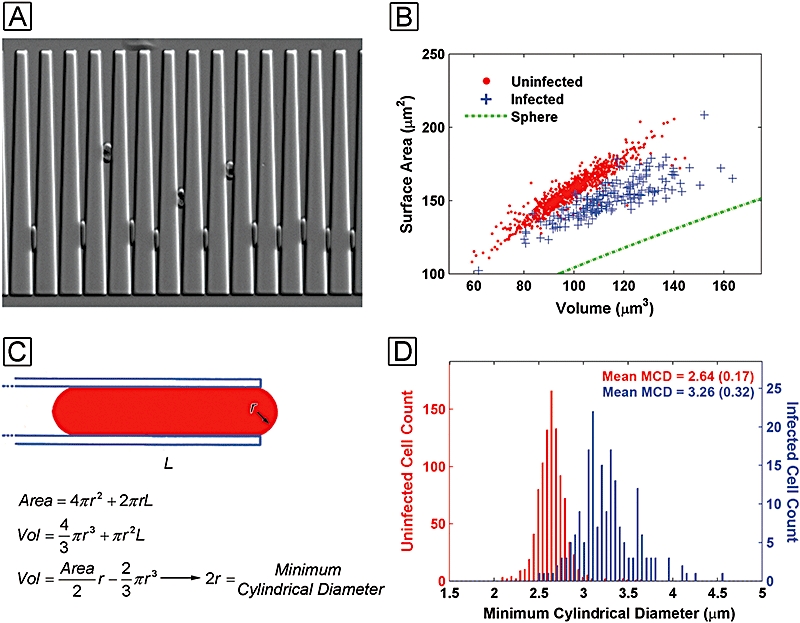
A. Schizont-IRBCs can easily be distinguished over the URBCs by the haem pigment. B. When measurements are made on roughly 1000 schizont-IRBCs and URBCs a surface area and volume scatter plot are generated to show a clear separation between the two cell populations. C. The MCD is found by solving a polynomial expression. D. A histogram of IRBCs and URBCs critical diameters shows that the IRBCs have mean MCD larger than that of the URBCs with the standard deviation of the population given in parentheses.

Microfluidic channels were fabricated using conventional photolithography and replica molding techniques (McDonald *et al*., 2000; Shelby *et al*., 2003). Individual RBCs were trapped in wedge-shaped microfluidic channels (Fig. 1A). The surface area and volume of an individual RBC were calculated by measuring the cell's length and position in the wedge (Gifford *et al*., 2003; 2006). Note that two different cells with the same volume but different surface area would settle into a wedge such that the cell with a larger surface area would penetrate farther into the wedge than the cell with the smaller surface area. Similarly, two cells with different surface areas and different volumes would end up settling to the same depth in wedges but would differ in their overall length of the cell. Data from thousands of individual RBCs were recorded by collecting multiple images of trapped cells over identical wedges arrayed throughout the microfluidic device.

Two *Plasmodium falciparum* strains were chosen for the present studies, the knob-forming adherent strain ITG and the knobless strain Dd2. Parasites cultures were synchronized using standard methods and then enriched to > 95% trophozoite-stage parasites using a magnetic chromatography column (Ljunstrom *et al*., 2004). Prior to a life-cycle time-course experiment, the enriched trophozoite parasites were suspended with fresh RBCs to avoid artifacts in URBCs from storage and long-term co-culture with parasitized RBCs. The cultures were then monitored at 6 h increments in order to follow schizont-infected cells, as they released their merozoites to invade RBCs and ultimately developed into new schizonts, as well as the fate of URBCs in the same culture.

Interpreting surface area and volume data requires a high level of confidence in the accuracy of these measurements. MCD measurements generally had a coefficient of variation of approximately 3% over repeated measurements of fresh blood samples. Additionally, two independent tests were performed to validate the microfluidic measurements. In the first test, independent of the trapping experiments in microfluidic wedges, malaria parasites were passed through a series of microfluidic flow channels with varying constrictions to identify the largest constriction that obstructed cell flow. This value was compared with the predictions made from surface area and volume measurements determined in the wedges. In a second test, parasite cultures were passed through 3 μm polycarbonate membrane filters. The MCD histograms before and after passage through the polycarbonate filter were compared. Changes in the shape and shifts in the mean values of the filtrate MCD histograms were compared with a predicted histogram derived from the product of the unfiltered MCD histograms and a filtration probability model.

### MCD of schizont-IRBCs

Comparing URBCs and IRBCs in microfluidic wedges illustrates how this technique can be used to gain surface area, volume and MCD measurements on a large population of individual cells. Schizont-IRBCs settled into much wider positions in the microfluidic wedges compared with fresh URBCs (Fig. 1A). In this image individual URBCs varied in the depth that they penetrated into the wedge-shaped constrictions indicating these URBCs surface areas and volumes were heterogeneous. The surface area and the volume of URBCs from a single donor varied by twofold across the entire population and appeared to have a linear relationship which has been observed in previous studies (Fig. 1B) (Canham and Burton, 1968; Gifford *et al*., 2003).

The Dd2 schizont-IRBC population exhibited both decreased surface area and increased volume compared with URBCs (Fig. 1B). The MCDs for individual cells were calculated by solving for the roots of a polynomial expression using each cell's surface area and volume (Fig. 1C). For the Dd2 schizont-IRBCs, both a decreased surface area and an increased volume contributed to an increase in the MCD of the infected cells over that of uninfected cells. A histogram of MCD measurements showed a clear separation between the schizont-IRBC population and the URBC population at this stage of parasite development (Fig. 1D). The increased MCD of the Dd2 schizont-IRBCs should make these cells more susceptible to filtration by the spleen, as is commonly accepted.

### MCD from ring-IRBCs to trophozoite-IRBCs

**Fig. 2:**
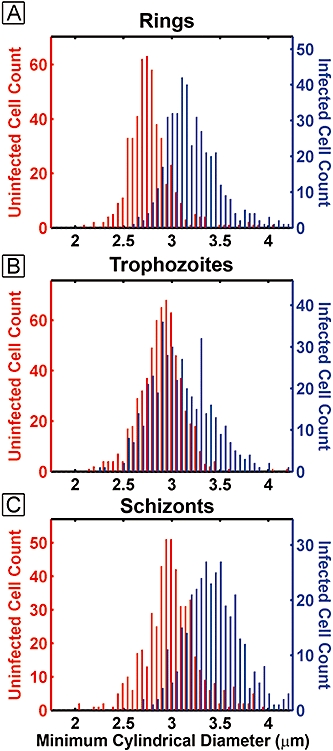
Histograms of critical diameters comparing co-cultured URBCs and IRBCs as they progress from ring (A) to trophozoites (B) and finally schizont (C). The trophozoite-infected MCD shows the greatest amount of overlap with the URBC MCD distribution. IRBCs are shown as the blue bars and URBCs are shown in red.

An increase in MCD of Dd2 ring-IRBCs was observed over that of URBCs (Fig. 2A). The MCD of these Dd2 ring-IRBCs was comparable to that measured from schizont-IRBCs, indicating that ring-IRBCs may be as likely to be retained in a filter as schizonts. As the parasites matured from rings to trophozoites, their MCD distribution decreased to overlap that of URBCs (Fig. 2B). The difference between trophozoites and rings at this point is notable because a larger percentage of cells have an MCD that is less than the ring stages. Dd2 schizont-IRBCs once again regain a larger MCD when compared with URBCs (Fig. 2C).

**Fig. 3:**
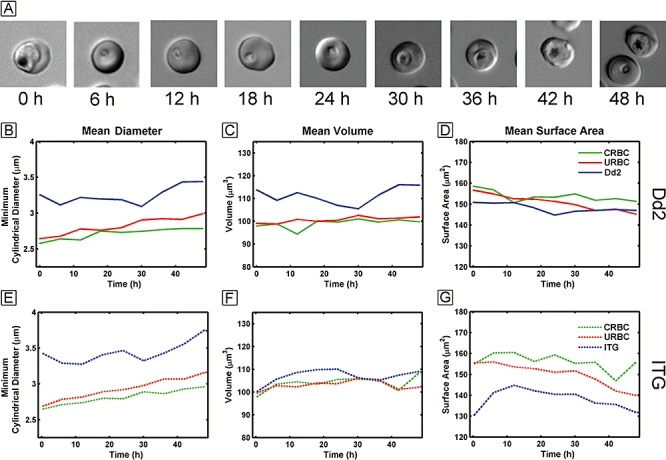
A. DIC images of parasites over the 48 h time-course. B–G. The mean diameter for Dd2 and ITG (B and E), the mean volume (C and F) and the mean surface area (D and G) are plotted for control-RBCs (green), IRBCs (blue) and URBCs (red) over the 48 h time-course.

To evaluate this result more closely, the development patterns of IRBCs, URBCs and control-RBCs cultured separately from parasites were compared. Two parasite strains, the adherent strain ITG and a non-adherent strain Dd2, were evaluated during two separate time-course experiments. Each experiment began at time 0 h with highly synchronous schizonts which were within 2–4 h of releasing their merozoites (Fig. 3A). These parasite cultures were evaluated in microfluidic wedges at 6 h increments over a 48 h time-course. As described above, surface area and volume were measured and the mean MCD was calculated from the measured values. Each data point represents a mean calculated from between 200 and 400 IRBCs and between 700 and 1000 URBCs. Variations in the mean MCD (Fig. 3B and E), the mean surface area (Fig. 3C and F) and the mean volume were recorded over the 48 h time-course (Fig. 3D and G). Plotting these values illustrated the relative contribution of each parameter towards fluctuations in the mean MCD.

At all time points, with both stains, the MCD of the IRBCs was always higher than that of URBCs (Fig. 3B and E). Unexpectedly, a decrease in IRBC's mean MCD was clearly visible as the parasites matured from rings to trophozoites from 6 to 30 h (Fig. 3B and E). Dd2- and ITG-IRBCs exhibited the lowest mean MCD at 8 h (rings stages) and at 30 h (trophozoites), indicating that from a strictly geometric standpoint, trophozoites could deform to a greater degree than schizont stages.

A breakdown of volume and surface area measurements indicated that, above all, the MCD of the Dd2-IRBCs is predominantly influenced by changes in volume of the parasitized cells as they mature (Fig. 3C). Dd2 rings at 12 h show a high MCD due to the increased volume of the IRBCs. Trophozoites at 30 h have the lowest volume of all other stages during parasite development which contributes to the lowest MCD for all parasite stages. When Dd2-IRBCs are compared with URBCs, Dd2-IRBCs surface area is reduced for all stages in the parasite life cycle. However, changes in surface area for Dd2-IRBCs are more subtle over the asexual life cycle. IRBCs initially loose membrane surface area during the first 24 h but thereafter steadily maintain it (Fig. 3D).

A similar, but not identical, development trend was observed for the adherent ITG-IRBCs. ITG-IRBCs did not show as large an increase in volume as Dd2 (Fig. 3F). At 30 h the ITG-IRBCs mean volume goes through a minimum which contributes to the MCD behaviour similar to Dd2. However, the changes in volume are not as pronounced in ITG-IRBCs as the volume changes in Dd2-IRBCs. Instead, ITG-IRBCs show large surface area drops that contribute to high MCD throughout the cycle. In particular, surface area losses from 12 to 48 h contribute to an increased MCD. The mean surface area of ITG-IRBCs is the smallest at schizont stage at 0 h and at 48 h which contributes to the large mean MCD at schizont stage. The differing methods in which the two strains, Dd2 and ITG, vary their MCD would be missed without applying the present techniques, which independently measure surface area and volume of each IRBC in a population.

### Co-cultured URBCs lose surface area

The MCD of URBCs in culture with both ITG- and Dd2-IRBCs increased steadily over time when compared with control-RBCs that had never been exposed to parasite cultures (Fig. 3B and E). However, unlike with IRBCs, the volume of the URBCs remained relatively constant (Fig. 3C and F). The steady increase in URBCs MCD was a result of a steady loss of mean surface area (Fig. 3D and G). Control-RBCs maintained at the same density as those in malaria culture also lost some surface area but at a lower rate than URBCs co-cultured with malaria parasites.

### Validation of MCD by blocking capillary constrictions

**Fig. 4:**
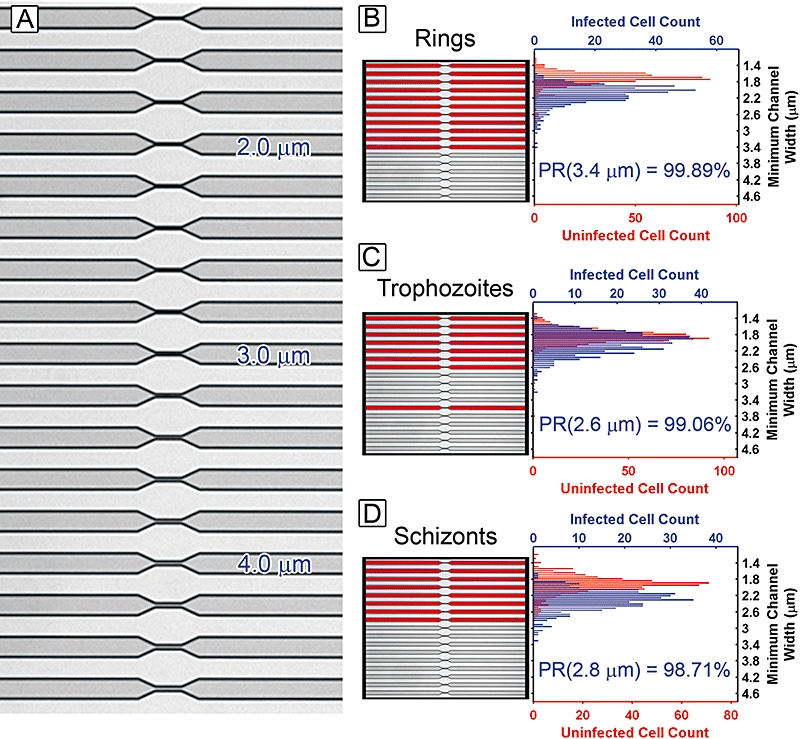
Channel constrictions with widths that range from 1.4 to 4.6 μm (A). Parasite cultures flowed through the constrictions at an applied pressure drop of 2.0 kPa for approximately 2 min. The channels shown in red had IRBCs or URBCs which blocked flow for each stage of rings (B), trophozoites (C) and schizonts (D). The width of the largest blocked channel had a percentile rank (PR) in the MCW histograms of 98–99% indicating that 1–2% of the cell in the culture had a MCW larger than that of the largest blocked channel.

The predictive value of surface area and volume measurements was evaluated by flowing parasite cultures through an array of microfluidic constrictions with varying widths (Fig. 4A). The largest constriction that trapped cells was evaluated at a physiologically relevant pressure of 2.0 kilopascals (kPa). Since the cross-section of the microfluidic constrictions was not circular, the altered constriction geometry required calculation of a minimum channel width (MCW) which is also based on the surface area and volume of each cell. The MCW of constrictions which became fully or partially blocked followed predictions made by MCW histograms (Fig. 4B–D). The largest constriction that a culture obstructed for each parasite stage corresponded to 98th to 99th percentile rank from the MCW population histogram for that stage. This means that 1–2% of the cells in the culture with the highest MCW determined the widest channel that was clogged by the trapped cells. At an applied pressure of 2.0 kPa, the largest channel blocked for a particular time point was predicted by the 99th percentile to within 0.2 μm. This predicted accuracy is within the tolerances of the photolithography tools used to generate the channel structures. As shown, the ring-IRBCs parasites actually obstruct larger channel width than the schizont-IRBCs. This could be due in part to rare double-infected RBCs that are a minor contributor to the MCD and do not survive as they mature trophozoite-stage parasites. Overall, these results show that surface area and volume measurements can be used to accurately predict a cell's ability to traverse narrow constrictions.

### MCD of bulk filtered RBCs

Microfluidic analysis tools were combined with bulk filtration to test whether MCD predicts selection between IRBCs and URBCs. The surface area and volume measurement, combined with MCD calculation, were used to characterize *P. falciparum* Dd2-IRBCs and URBCs before and after they had passed through filters with 3 μm pores. The filters were expected to remove Dd2-IRBCs as the filter pore diameter was lower than the MCD of these cells. As shown below, the MCD properties of individual cells could predict whether they would be removed by a filter with a defined pore size. However, RBCs which survived filtration did not emerge unchanged. The MCDs of URBCs became larger as a result of passage through 3 μm pores.

**Fig. 5:**
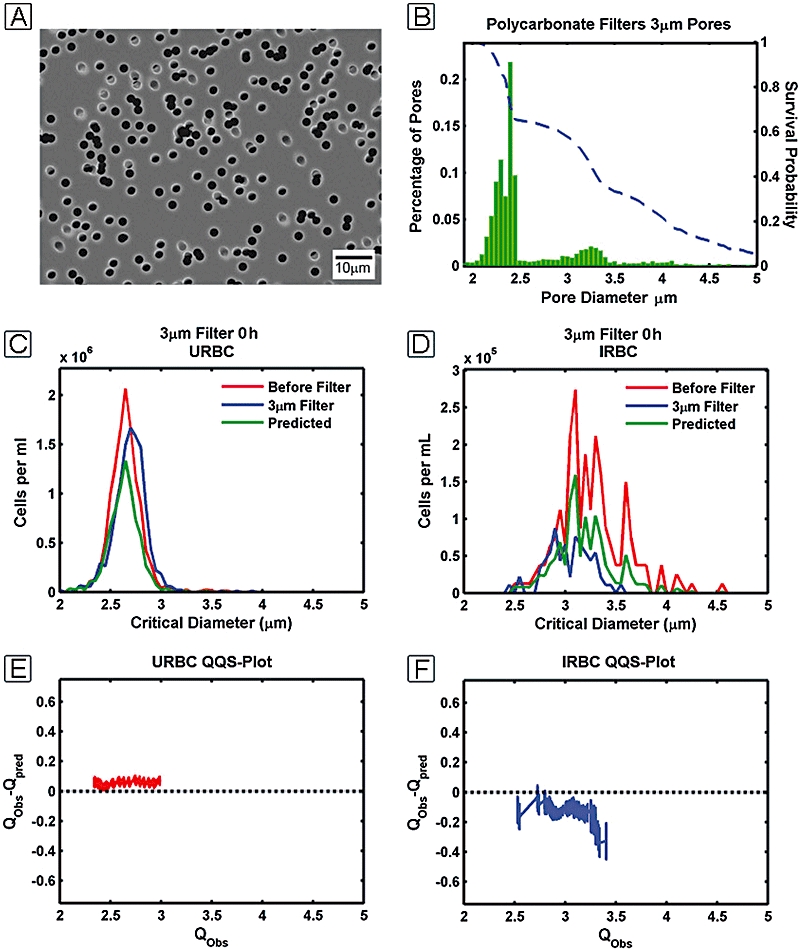
Cultures were passed through polycarbonate membrane filters (A) with a pore diameter distribution measured in the SEM (B). The blue line in (B) is the survival probability function of the filter calculated from the pore diameter distribution. The survival probability function was used to generate a predicted population distribution (C and D). The QQS plots for the URBCs (E) and IRBCs (F) are generated by plotting the quantiles of the observed distribution (*Q*_obs_) versus the difference between the quantiles of the predicted distribution and the observed distributions (*Q*_obs_ − *Q*_pred_) for the respective IRBC and URBC populations.

Predictions of cell filtration required reliable methods for measuring cell geometry and accurate measurements of the pores in the filter. Polycarbonate filters with well-defined pore sizes are commercially available but pores in these filters did not have a completely uniform size. Partial overlap of some pores presented opportunities for passage of cells with larger MCDs, cells that would normally not be able to get pass through a single 3 μm pore. Even though such overlapping pores were rare, they prevented a simple, direct comparison of RBC populations before and after filtration since larger cells were not completely eliminated. Instead, cells with MCD close to or above that of the filter pore size were attenuated as a function of their MCD. To develop a reliable filtration model that accounted for the variation in pore diameter, the exact pore diameter distribution of the polycarbonate filters was measured in a scanning electron microscope (Fig. 5A). Since flow rate through different pores varies by the fourth power of the pore diameter, the fractional amount of liquid that passed through each pore was calculated and used to derive a filtration survival probability function for a cell with a given MCD (Fig. 5B). The product of the survival probability distribution and the MCD distribution of any cell population predicted the MCD distribution of the filtered cell population, after passing through the membrane filter (Fig. 5C and D). A quantile–quantile shift (QQS) plot was used as a statistical tool for comparing how the predicted MCD distribution after filtration (derived from a sample population observed before filtration) differed from the actual sample population observed after passing through the polycarbonate filter (Fig. 5E and F). QQS plots compare both changes in the mean and changes in the shape of two sample population histograms.

The utility of the QQS plot is seen with data on URBCs (Fig. 5E). The plot compares the URBC distribution predicted using the survival probability function to the URBC distribution observed after passing through a 3 μm polycarbonate filter. The QQS plot has a near zero slope over its length but all of the plotted values are positive. The flat QQS plot indicates that the predicted MCD distribution has the same shape as the MCD distribution observed after the cells were filtered. Yet, the absolute MCD values of the filtered population increased by approximately 0.1 μm over what is predicted from the filter's survival function. The URBCs MCD became slightly larger after passing through the 3 μm polycarbonate filter. This phenomenon was also seen when the cells were passed through a much wider 5 μm polycarbonate filter (data not shown).

The QQS plot of Dd2-IRBC population showed the opposite effect. The infected cells that survived filtration had a smaller MCD than what was predicted (Fig. 5F). Since the entire culture of both Dd2-IRBCs and URBCs was filtered at the same time, the negative shift of the IRBCs must be due to the presence of parasites in the IRBCs. The negative slope of the IRBC QQS plot also points to a cell distribution that is slightly narrower compared with the unfiltered population, revealing the higher survivability of IRBCs with lower MCDs.

Overall, MCD calculations, derived from static cells in microfluidic wedges, can be an important approximation of whether a cell may actually pass through a pore or constriction of a defined size. However, calculations of MCD depend on accurate fabrication and calibration of the microfluidic devices and careful control of cell medium conditions during the experiments. MCD calculations do not take into account dynamic mechanical cell properties such as the viscoelasticity of a cell and strain rates at which the membrane can fracture. However, measurement of an MCD may be extrapolated to understand whether a cell's survival in circulation is dominated by cell geometry or by mechanical properties of related to deformability.

## Discussion

### The method

The present work adapts microfluidic devices to measure surface area and volume of IRBCs and URBCs and to determine how a cell's geometry may affect the ability of individual cells to survive filtration. In addition to demonstrating the robustness of microfluidic techniques and the value of making absolute measurements on large populations of individual cells, we specifically demonstrate how measurements on large populations of IRBCs and URBCs help predict the behaviour of cells when passed through filters and small constrictions.

Previous investigations on filterability of malaria cultures have shown that malaria infection does change the rheology of RBCs as measured by passing them through polycarbonate membrane filters (Miller *et al*., 1971). Also recent experiments have shown that IRBC's membrane stiffens as the infection progresses (Paulitschke and Nash, 1993; Glenister *et al*., 2002). However, the present study is the first to measure the geometry of thousands of individual cells, in terms of both surface area and volume, and to use these measurements to predict if the individual cells can pass through constrictions of a specified size or be segregated by physical filtration. This work relied on microfluidics as well as batch image analysis to allow for sampling of large populations of IRBCs and URBCs.

### Larger MCD of parasitized RBCs

Malaria parasites extensively modify the host RBC as the parasite invades, grows and matures within the host. These structural modifications are suspected to alter blood rheology, and can ultimately compromise circulation and lead to organ failure. This study investigated how surface area and volume of IRBCs and URBCs varied during the 48 h erythrocytic life cycle. While previous studies have investigated the forces required to deform a cell, MCD measurements provide insights into how the intrinsic limits of deformability can vary as parasites progress from ring stage to the schizont stage.

All infected RBCs had a larger average MCD than URBCs. In principle, depending on the cut-off criteria of the spleen, almost all parasitized RBCs should be filterable. For both the lab-adapted strains Dd2 and ITG, schizont-IRBCs had the largest MCD and therefore could deform the least amount compared with trophozoite-stage parasites. However, surprisingly, ring-infected RBCs had a high MCD, even higher than trophozoites. If the spleen is described strictly as a size-selective filter then both ring-IRBCs and schizont-IRBCs would be removed preferentially from circulation due to their larger MCD over that of URBCs. At first, this appears counterintuitive because circulating *P. falciparum* in patients are predominantly ring-IRBCs, even though trophozoites can have a lower MCD and are least likely to be filtered by the spleen (Baruch *et al*., 1999; Chen *et al*., 2000; Miller *et al*., 2002).

Why do ring-IRBCs dominate in circulation over other stages of parasites? The results presented here show that IRBCs and URBCs are not monodisperse and have a distribution of MCDs (Fig. 2A). The variation in MCD within populations of URBCs or within IRBCs can be as large as the difference between the average MCDs of these two populations. A small portion of the ring-IRBCs MCD histogram overlaps with the URBCs MCD histogram. The ring-IRBCs which are observed in peripheral circulation may have an MCD small enough to pass though the spleen and survive in circulation, before they go on to sequester and reproduce. If this explanation is true, ring-IRBCs observed in circulation may only be a subset of the total population because the spleen would have filtered cells with an MCD greater than that of URBCs. These views on fractional removal of ring-infected RBCs are consistent with previous data from *ex vivo* perfusion of human spleens, where only a fraction of ring-IRBCs were retained by splenic filtration and a small subset of the ring-IRBCs were observed not to be sequestered by the spleen (Safeukui *et al*., 2008). With these observations in mind, parasitaemia measured from peripheral circulation may not be a complete representation of the total number of ring-parasites, particularly since ring-IRBCs have been observed to cytoadhere (Baruch *et al*., 1996; Pouvelle *et al*., 2000; Douki *et al*., 2003). Our MCD data provide a more formal argument for why parasites may have evolved to express cytoadherence during the ring stages. The larger the fraction of rings-IRBCs which sequester and avoid filtration would lead to higher the parasite numbers in the patient.

### Larger MCD from larger volume

In Dd2 parasites, the MCD of IRBCs closely follows variations in their mean volume (Fig. 3B and C). The smaller MCD of trophozoite-RBCs can be attributed to a reduced volume at this stage of parasite development. The gradual volume loss may be due to digestion of haemoglobin in the parasitophorous vacuole for catabolic needs (Goldberg, 2005; Liu *et al*., 2006), particularly break-up of ‘the big gulp’ vesicle that first brings haemoglobin to the parasite cytoplasm (Elliott *et al*., 2008; Gligorijevic *et al*., 2008). As the parasites develop from the trophozoite stage into schizonts during 30–48 h, the parasites may increase in volume as they accumulate material for producing merozoite daughter cells. However Dd2 was observed to have a much larger volume increase than ITG. These differences in volume changes in parasitized cells may represent important biochemical variations in parasite metabolism, including management of haemoglobin degradation. In future, genetic dissection of such traits may allow more direct connection between parasite biochemistry and variations in deformability.

### Larger MCD from reduced surface area

All RBCs lost surface area while in culture, but the amount of surface area lost varied between IRBCs, URBCs and RBCs cultured without parasites present. The MCD increases can arise from a combination of variation in volume and decrease in surface area, particularly as seen with the strain ITG (Fig. 3E–G). The variation in surface area can come from the fact that parasites extensively modify the host RBC membrane (Bannister *et al*., 2004; Cooke *et al*., 2004; Smith and Craig, 2005; Gifford *et al*., 2006; Antia *et al*., 2007; Mills *et al*., 2007; Sens and Gov, 2007). For instance, parasites may maintain a constant surface area by adding components to the host membrane, as is seen in Dd2. However, in the case of the knob forming and adherent strain ITG, the membrane surface area of IRBCs is lost at a greater rate than Dd2-IRBCs. The limits on surface area restoration in ITG-IRBCs may be related to the presence of knobs and the underlying cytoskeletal links to the RBC. The loss of surface area in URBCs could also occur through one or more possible mechanisms. For example, phospholipids could be lost due to oxidative breakdown and hydrolysis. Alternatively, membrane components could be shed from RBCs by diffusion and equilibrating with the surrounding media. Since URBCs in culture with malaria parasites show a higher rate of surface area loss, this suggests that parasites may also scavenge membrane material from URBCs.

### Value of microfluidic tools for measuring MCD

The present results show phenotypic variations in lab-adapted parasite strains of *P. falciparum* in ways that are not easily seen by any other method. The MCD profiles of an adherent parasite strain ITG and a non-adherent strain Dd2 show subtle but significant differences. An important note is that both parasite strains depicted in this study have been lab adapted. The constant selection pressure by the spleen *in vivo* may alter the MCD profile of wild-type parasites. Clearly, one must be careful not to overestimate the behaviour of parasites which have been in culture for long periods of time. At the same time the work with laboratory strains of parasites illustrates that, in general, altering the MCD profile of IRBCs and URBCs can possibly influence the pathological progression of malaria infection by affecting the number of parasitized cells which are removed with each pass through the spleen.

Previous work has shown that MCD varies between individuals and varies with age (Canham and Burton, 1968). An individual's MCD distribution, then, may be related to filtration properties of that individual's spleen and may affect the progression of a malaria infection. The lower the spleens' MCD cut-off value, the higher the chance of capturing and removing all parasitized RBCs from circulation. Of course, the penalty would be a higher chance of removing more URBCs with a high MCD, possibly contributing to anaemia. Individuals who permit circulation of RBCs with a higher MCD may have lower anaemia but may allow more parasites to survive in circulation. The present tools are expected to help enormously in the way we evaluate variations in disease management between individuals in malaria endemic countries.

This combination of tools described here also allowed us to determine whether a population of individual cells deviated from a simple filtration model. In one example shown (Fig. 5E), the URBCs MCDs increased after passing through a 3 μm filter. Thus, these microfluidic measurements have unexpectedly detected a deformation-induced biological response in RBCs. The apparent increase in MCD might be related to deformation-mediated ATP release (Olearczyk *et al*., 2001; Sprung *et al*., 2002).

### Conclusions

Measurements of large populations of IRBCs and URBCs allow for predictions of these cells behaviour when they passed through filters and small constrictions. Such observation and modelling of flow of IRBCs and URBCs through filters and constrictions should lead to improved understanding of cell filtration by the spleen. These types of observations may also help determine the causes of variations in outcome of severe and complicated malaria cases in terms of splenic function and likely hood of capillary blockage. Finally, the present exploratory studies on geometric changes in infected cells point to possible importance of cytoskeleton-associated knobs in restricting the total volume of parasitized RBC, the possibly salvage of membrane components from uninfected RBCs, and pressure-induced changes in RBC geometries as they pass through narrow constrictions.

## Experimental procedures

### Culturing methods

Malaria cultures of *P. falciparum* strain Dd2 and ITG were cultured using standard techniques. Briefly, all parasites and RBCs were cultured under blood gas at 2% haematocrit in RPMI 1640 (Invitrogen) supplemented with 5 mg ml^−1^ Albumax I (Invitrogen) and 0.1 mg ml^−1^ hypoxanthine. The osmolarity of all growth media was adjusted using 10× phosphate-buffered saline to 300 mOsm as measured by a WESCOR vapour pressure osmometer. Cultures were grown to 8% parasitaemia and then synchronized 2× in 4 h with 5% d-Sorbitol. The cultures were allowed to progress through one invasion cycle and then the rings were synchronized again with 5% d-Sorbitol. After 24 h the parasites were highly synchronized in late trophozoite stages. These trophozoites were then isolated on a Midi MACS LS magnetic column (Miltenyi Biotec). The magnetic column enriched parasites to 99% purity. These trophozoites were diluted into RBCs to a parasitaemia of 10%. RBCs were obtained about 2–3 h earlier using venous puncture and citrate phosphate dextrose (Sigma) and washed to remove the duffy coat and plasma. The cultures were diluted to a density of 5 × 10^7^ cell ml^−1^ and 4 ml of cultures were placed in T-25 flasks tilted at 45°. The tilted flask concentrated the RBCs along the bottom edge of the flask so that even at low cell densities the cells were in close enough proximity for invasion to occur in the precipitated culture. A total of 10 flasks with 4 ml of culture were generated so that a single flask could be used for each time point. One flask was used for each time point because opening the flask for sampling and then gassing the flask with blood gas caused enough evaporation that the osmolarity of the media increased enough to effect volume measurements of individual cells. This technique of enriching and diluting in fresh RBCs ensured that all parasitized cells and RBCs were free of artefacts that accumulate during refrigerated storage. Parasite stage was verified by observing the time since invasion, and morphology of cells observed both from Geimsa-stained smears and under differential interference contrast (DIC) in the microfluidic channels.

### Microfluidic channel fabrication

Microfluidic channels were generated using photolithography and replica molding techniques (McDonald *et al*., 2000; Shelby *et al*., 2003). Channel patterns were designed using TurboCAD v10 (IMSI) and used to generate a quartz-chrome photo mask (PhotoSciences). The photo mask was then treated with an antiadhesion pellicle coating (SUSS Mask Pellicle Technology). The channel pattern was transferred from the photo mask to silicon wafers (Montico Silicon) using AZ-1512 photoresist (AZ Materials). Relief patterns were then etched into the wafers using the Bosche deep reactive ion etch process (Oxford Instruments ICP 380). The Bosch deep reactive ion etch anisotropically etches silicon 90° to the wafer surface. The etching creates a series of ridges where the wafer is protected by the photoresist. The etch depth and feature size on the silicon masters were characterized using optical profilometery (WYKO NT3300) and scanning electron microscopy (JEOL 7000 SEM). The silicon masters were vapour primed with the release agent (tridecafluoro-1,1,2,2,-tetrahydrooctly)-trichlorosilane (Gelest) in a vacuum desiccator overnight. The channels were generated by casting polydimethylsiloxane (Dupont Sylguard 184) replicas. The polydimethylsiloxane (PDMS) is a two-part elastomer that when mixed is pored over the relief pattern of the silicon master. The PDMS is cured at 70°C overnight and removed from the master. The casts are then trimmed to expose channels to the PDMS face and tubing connections punched with a blunt 20-gauge needle. The coverslips and PDMS channels were then exposed to an oxygen plasma of 10 W for 40 s (Harrik Plasma Cleaner PDC-001). When the PDMS channels and coverslips were brought into contact they irreversibly seal together. The negative space in the PDMS formed by the relief pattern in the silicon master is now the microfluidic channel. After sealing the channels were aged at room temperature for 3–5 days so that they would become sufficiently hydrophobic to prevent cells from sticking to the PDMS or glass surface.

### Microfluidic measurements

Channels were filled under high pressure with culture media. All experiments were imaged using a Nikon TE2000-S microscope (Nikon USA) equipped with a Prior motorized stage (Prior scientific), a Photometrics CoolSnap EZ CCD camera and Metamorph Imaging System software (Molecular Devices). Imaging of wedge constrictions was performed using DIC. Once the channel array was aligned with the stage axis, 100 μl of culture was applied to the face of the wedge array channel and the cells drawn in by reducing pressure at the array outlet. A constant pressure of 2.5 kPa was applied across the channels as monitored with a manometer (Spur Scientific). Approximately 1500 cells could be recorded in 80 images of cells trapped in the microfluidic wedges using the 40× DIC objective. Cell density of the sampled culture flask was also recorded using an improved neubauer haemocytometer.

After recording images from the microfluidic wedge array, cells were passed through an array of constrictions to observe the largest-size constriction that cells would obstruct. The channels were also filled under pressure and then 100 μl of sample was added to the channel inlets. Channels were imaged in bright field conditions using a 10× objective. A pressure drop of 2.0 kPa was applied to the channel for 2 min and then a Metamorph image stack was recorded for 10 s.

For bulk filtration experiments, a 1 ml portion of the culture was passed through 3 μm polycarbonate filters (Millipore Isopore™). The pressure drop across the filter assembly was maintained at a relatively constant pressure of no greater than 5 kPa by monitoring a manometer. After passing an aliquot of the culture through a polycarbonate filter, the filtrate was analysed using the microfluidic wedge system and the constriction array.

### Batch image analysis

A batch image-processing algorithm was developed using the MATLAB image-processing toolbox. The algorithm extracts length and position of cells in the DIC images. An image threshold level was first found to segregate the bright white lines to the left of the cells. The threshold method was developed based on the derivative of the between-class variance (Otsu, 1979; Lin, 2003). Once the image threshold was found, the top and bottom of the wedge constrictions were located, and a hough transform was used to identify resulting white lines. The breaks in the white lines correspond to the position of the cells in the microfluidic wedges.

### Calculation of shape of cell in wedge

Cell dimensions were calculated according to a geometric model of the cell shape in the wedge. The cell shape was estimated as the sum of a series of four isosceles trapezoids, a cylinder and two hemispheres. The hemispheres are used as the cell caps. Their diameter is determined by the width of the channel at the cell's top and bottom position in the channel. The cylinder makes up the cell's edge curvature along the length of the cell at the channel corners. The actual curvature is not known. There was no reliable method for solving for this curvature. Therefore, the edge curvature is assumed to be a constant radius of 0.05 μm. Surface area is corrected for measuring the surface area volume of cells swollen in increasingly hypotonic media. These cells were observed to have a linear relationship between the length and position in the wedge which the slope the linear correction did not vary based on position the initial position in the wedge. A correction was added to the surface area model so that cells maintained constant surface area as they were swollen in hypotonic media. Volume was observed to change in a manner consistent with previous observations so no correction to the volume shape model have been added (Savitz *et al*., 1964).

### Calculation of MCD and MCW

The cylindrical diameter for each cell was determined by finding the roots of the following equation (Canham and Burton, 1968):


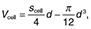


where *d* is the cylindrical diameter for an individual cell, *s*_cell_ is the cell surface area and *v*_cell_ is the volume of the cell. For solving for the MCW of a individual cell, the roots of the following equations were found (Canham and Burton, 1968):


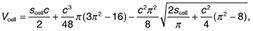


where *c* is the MCW for a cell of given surface area and volume. Solutions to both the cylindrical diameter and MCW were solved using the Newton–Raphson method.

### Calculation of survival probability function

The pore diameter of the polycarbonate sieves was used to develop a survival function for probability of particles of a certain diameter to pass through the sieve. The derivation of the survival function is as follows. The ferret diameters of membrane pores were measured by SEM. From these measurements a histogram distribution of the pore radii is generated. Flow rate *Q* though a pore is given by Poiseuille's equation:


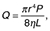


where *r* is the radius of the pore, *P* is the pressure difference across the *L* length of the pore and *η* is the viscosity of the fluid traversing the pore. The volumetric flow contributed by pores of a given size given by:


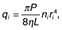


where *q*_*i*_ is the amount of flow given by the number of pores *n*_*i*_ of a given radius *r*_*i*_. Since the viscosity, pore length and pressure are identical for each pore in the polycarbonate sieve, the relative flow rate through each pore varies by *r*^4^. Then the survival function is written as:


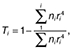


where *T*_*i*_ is the survival probability for a particle of radius *r*_*i*_. The survival function is plotted with respect to the histogram of pore diameters (Fig. 5B). This model assumes the following: pores do not clog, flow through pores is independent of time and cells that flow through the pores do not interact with each other.

### Calculation of QQS plots

In order to relate the measurements of cell distributions both before and after passing them through a polycarbonate sieve, the cell density and parasitaemia were measured. The observed cylindrical diameter distribution was generated from each culture and scaled by the cell density and parasitaemia to yield the total number of infected cells per ml. The predicted distributions were the product of the survival probability function and the observed uninfected or infected population. The predicted distributions needed to be randomly sampled using the bootstrap technique *in silico* which created a bootstrap population. The quantiles of the bootstrap population may then be compared with the quantiles of the observed population. The bootstrap population is a Monte Carlo simulation where a random sample population is generated from the predicted distribution. For the data analysis performed here, 1000 bootstrap populations were generated, the quantiles averaged and the standard deviations calculated. A QQS plot is then generated where the quantiles of the observed distribution were then plotted against the difference between the mean quantiles of the bootstrap population and the quantiles of the observed distribution.
